# Effects of unsaturated fatty acids on weight loss, body composition and obesity related biomarkers

**DOI:** 10.1186/1758-5996-7-S1-A139

**Published:** 2015-11-11

**Authors:** Vanessa Chaia Kaippert, Marcelly Cunha Oliveira dos Santos Lopes, Pires Denise de Carvalho, Eliane Lopes Rosado

**Affiliations:** 1 UFRJ-Universidade Federal do Rio de Janeiro, Rio de Janeiro, Brazil

## Background

Obesity is multifactorial disease that may be related to the development of comorbidities such as type 2 diabetes mellitus and cardiovascular disease.

## Objectives

Evaluate the effects of modulation of dietary polyunsaturated fatty acid (PUFA) and monounsaturated fatty acid (MUFA) on weight loss, body composition and obesity related biomarkers.

## Materials and methods

A parallel, randomized, controlled, single-blind study was conducted with dietary intervention (DI) for 60 days, which 32 women with class I and II obesity were distributed into three following groups: G1=normocaloric diet high in n-3/n-6 PUFA (12% of total energy intake, 10% of n-6 and up to 2% of n-3) (n=10); G2=normocaloric diet high in MUFA (15% to 20% total energy intake) (n=11); and G3=control group with maintenance of habitual diet (n=11).The diets prescribed for G1 and G2 had similar macronutrient composition, varying only the type of fats offered. For complementation of dietary fats were used soy and fish oils to G1 and olive oil to G2, which all groups received capsules and sachets containing oils or placebo. Anthropometric measurements, body composition (bioelectrical impedance analysis) and laboratory variables (glucose, insulin, free fatty acids (FFA), glycerol, adiponectin and leptin) were carried out before and after the DI. The insulin resistance and sensitivity were assessed by HOMA-IR (Homeostasis Model Assessment) and QUICKI (Quantitative Insulin Sensitivity Check Index) methods, respectively.

## Results

Before the DI, the variables did not differ between groups. All groups had similar caloric and macronutrients intake during the DI, differing only in the quality of dietary fats. G2 decreased body weight (-1.92 + 1.99 kg), body mass index (-0.69 + 0.70 kg/m2), waist circumference (-1.91 + 1.82 cm) and body fat mass (-1.14 + 1.53 kg). There were no effects on glycemic profile as well as the concentrations of FFA, glycerol, adiponectin and leptin. However, G3 showed increased insulin resistance.

## Conclusion

Diet high in MUFA promoted benefits on weight loss and body composition in women with obesity. There was no influence of the type of dietary fat in obesity related biomarkers.

